# Multi-Residue Analysis of Chemical Additives in Edible Vegetable Oils Using QuEChERS Extraction Method Followed by Supercritical Fluid Chromatography

**DOI:** 10.3390/molecules27051681

**Published:** 2022-03-03

**Authors:** Yaping Gan, Yan Zhu

**Affiliations:** 1 Ecology and Health Institute, Hangzhou Vocational & Technical College, Hangzhou 310018, China; ganyaping8308@163.com; 2 Department of Chemistry, Xixi Campus, Zhejiang University, Hangzhou 310028, China

**Keywords:** antioxidants, photoinitiators, UV absorbers, plasticizers, edible vegetable oils, QuEChERS, supercritical fluid chromatography

## Abstract

Since the quality and safety of food highly depend on its preservation and protection, the use of food packaging materials increases the risk of chemical contamination of the packaged food by migration. Herein, we focused on antioxidants, photoinitiators, UV absorbers and plasticizers which are extensive additives used in food packaging materials. In the present study, a rapid, simple, green and reliable method was developed and validated for the determination of twelve chemical additives in edible vegetable oils using SFC together with a modified QuEChERS procedure. Under the optimum conditions, twelve additives were separated within 10 min, and the consumption of the organic solvent was significantly reduced, which improved the environmentally friendliness. The performance of the developed method was evaluated. Good linearity (r > 0.999) was obtained in the range of 0.20–20.0 µg/mL and 0.50–20.0 µg/mL, respectively. The limits of detection and limits of quantification of the twelve additives in vegetable oils were 0.05–0.15 µg/mL and 0.15–0.50 µg/mL, respectively. Recoveries of all the chemical additives for the spiked samples were between 60.9% and 106.4%, with relative standard deviations (RSD) lower than 9.9%. The results demonstrated that the proposed method was efficient, reliable and robust for the routine analysis of additives in edible vegetable oils and can be an alternative to the multi-residue analysis of chemical additives for other packaged foods.

## 1. Introduction

Since the quality and safety of food highly depend on its preservation and protection, the use of food packaging materials increases the risk of chemical contamination of the packaged food by migration [1]. During the manufacturing of packaging materials, several kinds of additives, such as stabilizers, plasticizers, antioxidants and ultraviolet (UV) absorbers, are frequently introduced to improve the properties of the products [2]. However, chemical residues or additives released from packaging materials can cause foodstuffs contamination and even potential hazards, owing to the migration during the long-term direct contact process [3]. Therefore, evaluating the safety of foodstuffs exposed to packaging materials is an inevitable trend.

Herein, we focused on antioxidants, photoinitiators, UV absorbers and plasticizers which are extensive additives used in food packaging materials. Adding antioxidants can improve durability [4] and prevent degradation and aging of packaging materials. Tert-butylhydroquinone (TBHQ), butylated hydroxyanisole (BHA) and butylated hydroxytoluene (BHT) are the most extensively used synthetic antioxidants due to their high antioxidant capacity [5]. Photoinitiators (PIs) can initiate a polymerization reaction and cross-link curing under UV light [6,7]. However, typical PIs such as methyl 2-benzoylbenzoate (OMBB), 2,2-dimethoxy-2-phenylacetophenone (BDK) and 4-methylbenzophenone (4-MBP) have been found to be carcinogenic and have allergenic effects and reproductive toxicity [8]. Plasticizers are also widely used in packaging materials to improve flexibility and workability. Since these additives are not chemically bound to polymers, they can easily be released from the packaging materials into foodstuffs during the production and storage process, particularly under heat, pressure and contact with fatty or oily food [9]. It has been reported that high exposure to plasticizers such as benzyl butyl phthalate (BBP), bis (2-ethylhexyl) phthalate (DEHP) and trioctyl trimellitate (TOTM) may cause potential damaging effects on the kidneys, liver and lungs [10]. 2-hydroxy-4-methoxybenzophenone (UV-9), 2,2′-dihydroxy-4-methoxy benzophenone (UV-24), octabenzone (UV-531) and 2-(2-hydroxy-5-methylphenyl)benzo triazole (UV-P) are typical UV absorbers which are commonly used to prolong the stability and lifetime of materials since they can effectively prevent the degradation, such as yellowing, caused by solar radiation [11,12]. Similar to the previous additives, UV absorbers also have biotoxicity and can disturb the endocrine system [13,14,15]. To date, many institutes at home and abroad have issued regulations, standards or licensing lists to restrict the use of additives in food contact materials. The National Food Safety Standard—Application Standard for Additives Used in Food Contact Materials and Articles (GB 9685-2016) [16] stipulates the maximum level, maximum permitted (QM) and specific migration limit (SML) of various additives used in food contact materials. According to this standard, the SML of BHA is 30 mg/kg, and DEHP is 1.5 mg/kg. The Union Guidelines on Regulation (EU) No 10/2011 [17] on plastic materials and articles intended to come into contact with food established an overall migration limit (OML) for 4-MBP and a BP of 0.6 mg/kg.

Edible vegetable oils, which are abundant in unsaturated fatty acids and other nutrients, are widely used in daily diets. Packaging materials contaminated with harmful chemical constituents and the migration of such chemical additives from packaging materials are the main reasons for the occurrence of contaminants in edible vegetable oils (e.g., antioxidants, photoinitiators, UV absorbers and plasticizers). Therefore, developing sensitive and reliable methods to analyze these chemical additives is necessary for the quality control of vegetable oils. However, sample pretreatment is a tricky subject, since target compounds are fat-soluble and tend to remain in the fatty food sample matrix. To date, using acetonitrile to extract analytes from the oil matrix is one of the most reported methods as the first step of preprocessing [18,19,20]. Usually, extraction needs to be combined with clean-up to achieve satisfactory results. Gel permeation chromatography (GPC) was used frequently, but it is solvent-consuming and time-consuming [21]. Moreover, several studies applied solid phase extraction (SPE), solid phase microextraction (SPME) and dispersive micro solid phase extraction (DMSPE) to minimize the sample treatment steps for contaminant analysis in edible oils [22,23,24]. The quick, easy, cheap, effective, rugged and safe (QuEChERS) method developed by Anastassuades et al. [25] has received considerable attention due to its advantages such as simplicity, low solvent consumption and flexibility. This methodology involves liquid partitioning with acetonitrile and then purifying by dispersive solid-phase extraction (d-SPE). Nowadays, the QuEChERS method has been applied for the determination of bisphenols in milk [26], pesticides in edible vegetable oils [27], hymexazol in plant derived foods [28] and so on. Based on previous research, several attempts were made in this study to modify the above method as a pre-treatment for edible oils.

Most of the present analytical methods for the additives used in packaging materials are based on gas chromatography (GC) [29], liquid chromatography (LC) [30], gas chromatography-mass spectrometry (GC-MS) [31] and liquid chromatography-mass spectrometry (LC-MS) [32]. All the methods mentioned above have their respective merits, but overall, the consumption of organic reagents is relatively large and the analysis time is long. To date, supercritical fluid chromatography (SFC) has caused increasing public attention for its unique advantages, including that it is green, time-saving and highly efficient. Supercritical fluid with low viscosity is used as the main mobile phase in this methodology to achieve fast separation and high resolution at the same time [33,34,35]. Yue Qiu et al. [36] reported an SFC method for the determination of UV absorbents in plastic food contact materials. Yun Zhang et al. [37] applied SFC-MS/MS to evaluate the photoinitiators from food packaging.

The objective of this study was to develop a simple, rapid and reliable analytical method combining QuEChERS with SFC for the simultaneous analysis of twelve chemical additives (two antioxidants, three photoinitiators, three plasticizers and four ultraviolet absorbers, their information are presented in Table 1) in edible vegetable oils. The QuEChERS conditions, including sorbent types, amounts and anhydrous magnesium sulfate dosage, were investigated to achieve a satisfactory purification and extraction effect. The modifiers, flow rate, column temperature, backpressure and gradient procedure were optimized to separate different kinds of additives with a good resolution. The final method was applied for the qualitative and quantitative analysis of twelve chemical additives in various edible vegetable oils.

## 2. Results and Discussion

### 2.1. Optimization of QuEChERS Method

**Figure 1:**
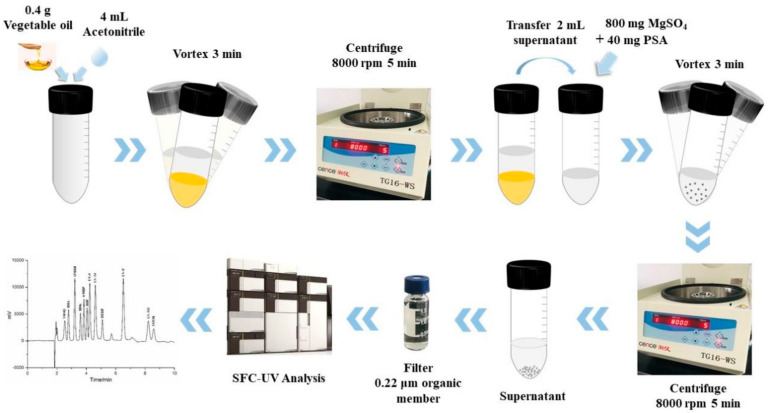
Schematic diagram of the QuEChERS process, followed by SFC.

For the determination of various additives in edible vegetable oil, the QuEChERS method involved two steps (as shown in Figure 1): liquid–liquid extraction (LLE) of 0.4 g edible vegetable oil with 4 mL acetonitrile, followed by salting-out with anhydrous magnesium sulfate; and dispersive-solid-phase extraction with sorbents. In this method, acetonitrile was chosen as the extract solvent because its lipid solubility is weak. However, some interferences, such as triglycerides, pigments, fatty acids and so on, can be transferred into acetonitrile from the oils, simultaneously. Hence, after LLE, ultracentrifugation was performed to remove the triglycerides, while the pigments, fatty acids and other co-extracts were further cleared by d-SPE. PSA, GCB and C18 are widely used as dispersive cleaning sorbents, and anhydrous MgSO_4_ is added to remove excess water. Specifically, PSA is a typical sorbent for the removal of polar interferences such as some sugars, polar pigments and organic acids. GCB can effectively remove pigments and sterols. C18 is used to adsorb the nonpolar impurities.

**Figure 2:**
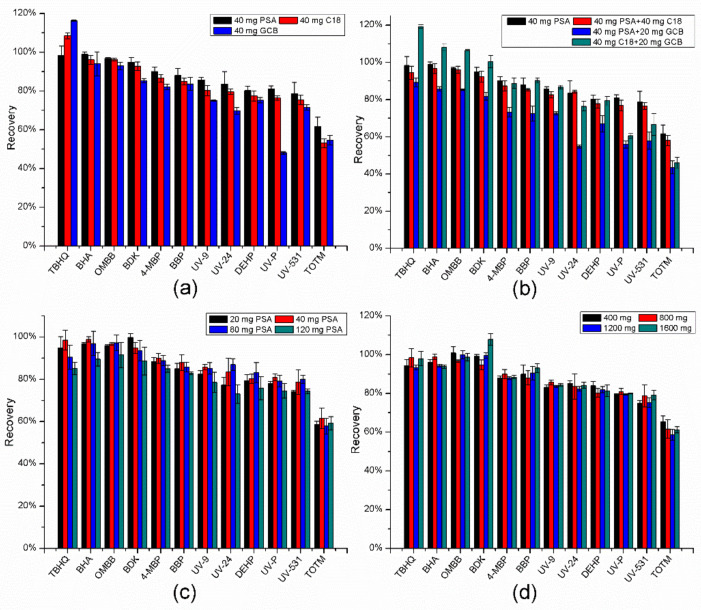
Optimization of the QuEChERS method. (**a**) Comparison of different sorbents. (**b**) Comparison of different hybrid sorbents. (**c**) Selection of the amount of PSA. (**d**) Selection of the amount of MgSO_4_. The other pretreatment conditions were as described in Section 3.3. Error bars mean standard deviations.

The first study was based on evaluating the clean-up effect of different sorbents (PSA, GCB, C18) in spiked blank oil samples (10 µg/g). Other pretreatment conditions were as described in Section 3.3, “Sample collection and preparation”. As shown in Figure 2a, better recovery value was observed with the PSA sorbent than C18 or GCB. When GCB was used as a sorbent, the recovery of TBHQ was too high, while UV-P and TOTM were less than 60%. In general, the PSA sorbent is a better choice for all target compounds. Since many studies which combined two or three sorbents in the d-SPE clean up steps have been reported, further exploration was done by trying different hybrid sorbents [38,39,40]. More specifically, the three sorbent combinations were as follows: 40 mg PSA with 40 mg C18, 40 mg PSA with 20 mg GCB and 30 mg C18 with 20 mg GCB. The results are shown in Figure 2b. It was quite clear that the recoveries of PSA combined with GCB were the poorest. As for the mixture of C18 and GCB, some recoveries were too high or too low. In addition, a comparison of the recoveries obtained from PSA alone and combined with C18 were not significantly different, or rather, the use of PSA alone was relatively better. Consequently, PSA was selected as the optimal sorbent for subsequent experiments.

After determining the type of d-SPE sorbent, the dosage also needed to be investigated. Herein, the various amounts (20, 40, 80 and 120 mg) of PSA were determined. When the dosage of PSA increased to 120 mg, recovery was lower (59.2–91.5%), since excess PSA can adsorb some target compounds, as shown in Figure 2c. Most of the chemical additives achieved the best recoveries (61.6–98.9%) when 40 mg PSA was used, except BDK, UV-24, DEHP and UV-531. Considering the cost of sorbent and recovery value, 40 mg PSA was used as the final cleaning sorbent to remove impurities.

In the QuEChERS process, anhydrous MgSO_4_ is often used as a dehydrating agent, because it has a high affinity for water and the salting-out effect. Dehydrating agents are usually added twice in conventional methods: the first is in the solvent extraction, and the other is together with the d-SPE sorbents. In this study, since there was very little water in the edible oil, the first step of dehydration was omitted. In other words, anhydrous MgSO_4_ was only added to the acetonitrile extract together with 40 mg PSA. As can be seen from Figure 2d, the amount (400, 800, 1200 and 1600 mg) of MgSO_4_ had no obvious impact on recoveries, probably because very little water existed in the acetonitrile extract itself. To be clear, five additives including TBHQ, BHA, 4-MBP, UV-9 and UV-P achieved the highest recoveries in 800 mg MgSO_4_ conditions.

### 2.2. Optimization of SFC Conditions

In order to improve the separation effect of twelve target additives, several SFC conditions, including the modifier, flow rate, column temperature, backpressure and gradient eluting procedure, were discussed, respectively. A Thermo Scientific^TM^ Acclaim^TM^ 120 C18 (5 µm, 4.6 mm × 250 mm) column was chosen. The wavelength of the UV detector was kept at 220 nm.

#### 2.2.1. The Influence of Organic Modifiers

**Figure 3:**
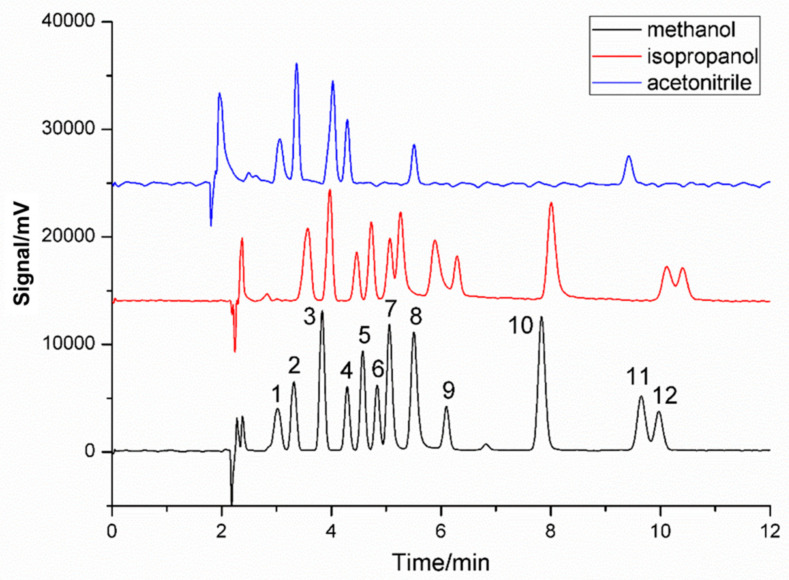
Effect of different modifiers. Column temperature: 40 °C; BPR: 10 MPa; flow rate: 1.6 mL/min; UV wavelength: 220 nm; Peak: (1) TBHQ, (2) BHA, (3) OMBB, (4) BDK, (5) 4-MBP, (6) BBP, (7) UV-9, (8) UV-24, (9) DEHP, (10) UV-P, (11) UV-531, (12) TOTM.

The application of SFC is limited by a supercritical CO_2_ eluent with low polarity. Therefore, the addition of a polar organic co-solvent, always called a modifier, is essential to adjust the eluent strength and enhance the solubility of the target analytes. Organic modifiers, including methanol, isopropanol and acetonitrile, were introduced into the mobile phase to improve the separation of the twelve target compounds on the C18 column. As shown in Figure 3, the acetonitrile exhibited obviously poor peak shapes, and many analytes were co-eluted compared to the methanol and isopropanol. This was probably because acetonitrile lacks the capacity of the hydrogen bond donor, which could compete with the additives to interact with the functional sites on the column. As for the other two alcohol modifiers, methanol achieved more symmetric peak shapes, better separation and less analysis time, possibly because the polarity and hydrogen bond donor capacity of the methanol was stronger than the isopropanol. Additionally, the high viscosity of isopropanol may easily cause excessively high pressure. As a consequence, choosing the appropriate modifiers was indispensable for multiple target analyte separation, and methanol was adopted as the modifier for subsequent experiments.

**Figure 4:**
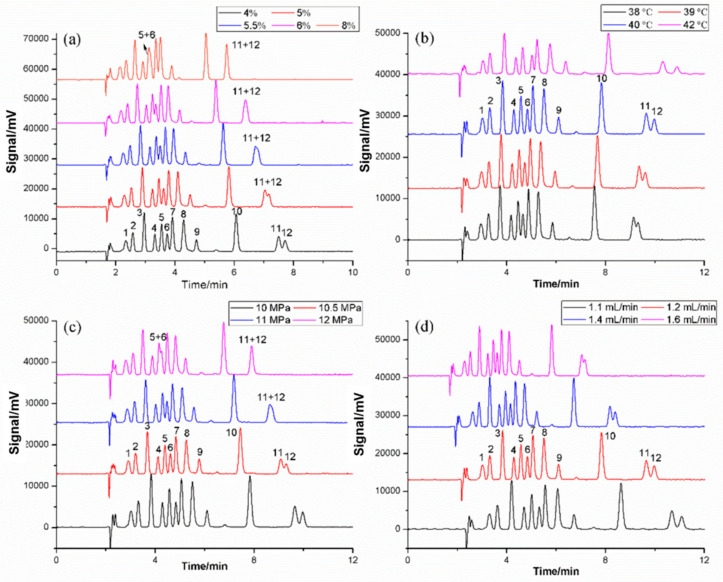
(**a**) Selection of the percentage of CH_3_OH. Column temperature: 40 °C; BPR: 10 MPa; flow rate: 1.6 mL/min. (**b**) Selection of column temperature. Modifier: 5% CH_3_OH; BPR: 10 MPa; flow rate: 1.2 mL/min. (**c**) Selection of backpressure. Modifier: 5% CH_3_OH; Column temperature: 40 °C; flow rate: 1.2 mL/min. (**d**) Effects of flow rate. Modifier: 5% CH_3_OH; Column temperature: 40 °C; BPR: 10 MPa. UV wavelength: 220 nm. Peak: (1) TBHQ, (2) BHA, (3) OMBB, (4) BDK, (5) 4-MBP, (6) BBP, (7) UV-9, (8) UV-24, (9) DEHP, (10) UV-P, (11) UV-531, (12) TOTM.

The proportion of the modifier is the main factor in changing the polarity of the mobile phase, leading to a different retention and separation behavior. In this research, a low content of the organic modifier (methanol from 4% to 8%) was added to acquire a better separation efficiency. As depicted in Figure 4a, a shorter retention time and better peak shape were acquired with the increment of the methanol proportion; however, the resolution of peak 5/6 and peak 11/12 tend to deteriorate and even overlap. Combined with peak shape and response, 5% methanol was selected temporarily for subsequent optimization experiments, although the separation in 4% was slightly better than in 5%.

#### 2.2.2. Effects of Temperature

Column temperature can affect retention behavior and separation efficiency via influencing the density and viscosity of the supercritical fluid. In this study, different temperatures (38, 39, 40, and 42 °C) were investigated. As shown in Figure 4b, the retention times of all target compounds were significantly prolonged with a temperature increase, as previously mentioned. This result was consistent with the previous description. Moreover, the resolution of UV-531 (peak 11) and TOTM (peak 12) improved when the temperature increased. However, an excessive temperature (over 40 °C) would affect the peak spread and peak symmetry, and also led to a worse resolution of BBP (peak 6) and UV-9 (peak 7). In summary, 40 °C was chosen as the final temperature in subsequent experiments.

#### 2.2.3. Effects of Backpressure

In SFC, backpressure (BPR) also has a significant impact on retention and separation behavior. Increasing BPR usually shortens the retention time and influences the separation effect attributed to the higher density and greater eluting strength of supercritical CO_2_. Different backpressures (10, 10.5, 11 and 12 MPa) were tested to get better separation in this study. From Figure 4c, an obvious result was that the retention time of all twelve compounds reduced with the increasing backpressure. However, when the backpressure elevated over 10 MPa, UV-531 (peak 11) and TOTM (peak 12) tended to overlap. At 12 MPa, even 4-MBP (peak 5) and BBP (peak 6) could not baseline separate. Therefore, on the basis of the abovementioned analysis, 10 MPa was the most reasonable backpressure applied in this research.

#### 2.2.4. Effects of Flow Rate

Flow rate is one of the optimization parameters in SFC. Apparently, a high flow rate can shorten the analysis time due to the density of the mobile phase increases. Compared with liquid chromatography, SFC can afford a higher flow rate due to the unique properties of supercritical fluid. The results of the different flow rates (1.1, 1.2, 1.4 and 1.6 mL/min) were shown in Figure 4d. The analysis time reduced from 12 to 8 min while the flow rate increased from 1.1 to 1.6 mL/min. However, when the flow rate exceeded 1.2 mL/min, UV-531 (peak 11) and TOTM (peak 12) could not separate well. As for the other ten target analytes, baseline separation was achieved with a flow rate from 1.1 to 1.4 mL/min. If the flow rate kept speeding up, it could be found that the resolutions of 4-MBP (peak 5), BBP (peak 6) and UV-9 (peak 7) would tend to deteriorate. If the flow rate was too slow, not only would the analysis time be longer but also the peak shape would become worse.

**Figure 5:**
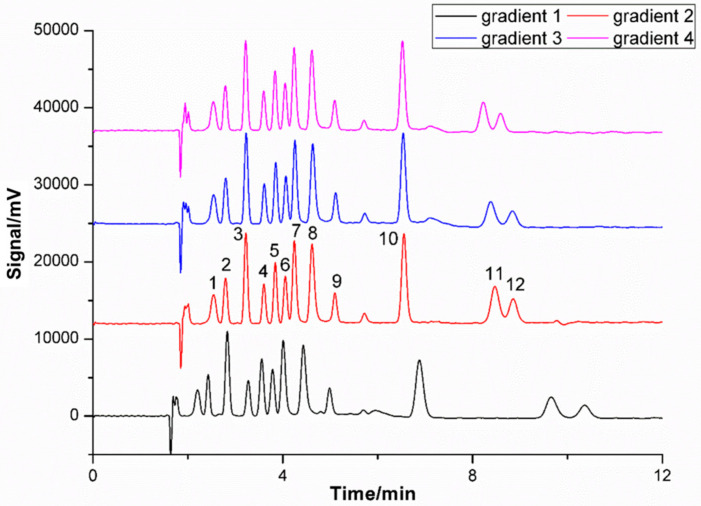
Comparison of different gradient elution programs. Column temperature: 40 °C; BPR: 10 MPa; UV wavelength: 220 nm; Peak: (1) TBHQ, (2) BHA, (3) OMBB, (4) BDK, (5) 4-MBP, (6) BBP, (7) UV-9, (8) UV-24, (9) DEHP, (10) UV-P, (11) UV-531, (12) TOTM.

According to the above comprehensive analysis of the modifier, column temperature, backpressure and flow rate, the gradient elution program was further explored to obtain the optimal separation effect. In this research, both the flow rate gradient and concentration gradient were optimized. The results of four different gradient elution programs were shown in Figure 5. Taking into account resolution, retention time and peak shapes, gradient 4 was finally adopted as the elution. The program was specifically as follows: 0–5 min, 5% CH_3_OH (95% CO_2_); 5–10 min, 3% CH_3_OH (97% CO_2_). As mentioned in Section 2.2, “Optimization of SFC conditions (effects of flow rate)”, the first ten target analytes were baseline separated with a flow rate from 1.1 to 1.4 mL/min at a concentration of 5% CH_3_OH. Hence, 1.4 mL/min was selected to shorten the analysis time. However, UV-531 (peak 11) and TOTM (peak 12) were not perfectly separated under such conditions. This problem was solved by changing the methanol concentration from 5% to 3% in the fifth minute, because the eluent strength of the mobile phase weakened with a lower modifier concentration. The optimal elution gradient conditions and other SFC conditions were presented in Section 3.2.

### 2.3. Method Validation

#### 2.3.1. Linearity, LOD and LOQ

A series of mixed matrix matched standard solutions (0.20, 1.0, 2.0, 5.0, 10.0, 15.0 and 20.0 μg/mL for BHA, OMBB, 4-MBP, UV-9, UV-24, UV-P; and 0.50, 1.0, 2.0, 5.0, 10.0, 15.0 and 20.0 μg/mL for TBHQ, BDK, BBP, DEHP, UV-531, TOTM) were prepared to establish the matrix-matched calibration curve. Satisfactory linearity was obtained for the twelve additives, with all correlation coefficient (r) values higher than 0.999. Spiked samples were prepared by spiking standards into blank natural samples and were used to determine the limit of detection (LOD, S/N = 3) and limit of quantification (LOQ, S/N = 10) values. The LODs and LOQs values of the twelve additives were calculated to range from 0.05–0.15 μg/mL and 0.15–0.50 μg/mL, respectively. All the results were summarized in Table 2.

#### 2.3.2. Matrix Effects

Compared to conventional pretreatment methods such as solid phase extraction, QuEChERS is much greener and simpler. Nevertheless, some impurities may be co-extracted during the QuEChERS process to cause matrix interference and finally affect the accuracy of results. Considering the mentioned conditions, the matrix effects (ME) were studied to investigate the influence of the substrate. The equation was as shown below and the values were listed in Table 2. Based on this experiment’s results, signals of some analytes were suppressed and others were enhanced. The values were between −4.30% and +6.70%, indicating that the matrix effects on the qualitative and response signals of target compounds were not obvious under the developed method. However, to ensure the accuracy and reliability of the final results, matrix-matched calibration curves were used to compensate for the influence caused by the vegetable oil samples.

$$\mathrm{ME}\;=\;\left(\frac{slope\;obtained\;from\;matrix\;matched\;standard}{slope\;obtained\;from\;solvent-based\;standard}-1\right)\times 100\%$$

#### 2.3.3. Accuracy and Precision

The recovery experiments and relative standard deviation (RSD) were investigated by spiking blank natural samples in three levels (1 µg/g, 5 µg/g and 10 µg/g) with six replicates to evaluate the accuracy and precision, respectively. As presented in Table 3, ten polymer additives had mean recoveries ranging from 81.0% to 106.4%, and two had recoveries varied from 60.9% to 79.9%. Meanwhile, the relative standard deviation (RSD, *n* = 6) of all recovery experiments were lower than 9.9%, showing the satisfactory precision of this method. Herein, the recoveries of the two target analytes were below 80%, which may be attributed to the higher molecular weight and lower polarity.

### 2.4. Application to Natural Samples

Considering that the polymer additives used in packaging materials may migrate into foodstuff, strict quality control and testing is essential to ensure the health of consumers. To further verify the applicability, the proposed method was applied to various natural samples, including two extra virgin olive oil samples, two peanut oil samples, two blend oil samples and one sunflower oil sample. Each sample was analyzed in triplicate and the results are summarized in Table 4. BHA was detected in extra virgin olive oil sample 2, with a concentration of 8.21 µg/g. OMBB was detected at 4.04 µg/g in blend oil sample 1, and another blend oil was found to contain 4.47 µg/g of BDK. Furthermore, other tested samples were found to be free of the twelve polymer additives’ contamination (<LOD). It was noticed that some types of polymer additives may migrate from packaging materials to vegetable oil in some situations. Therefore, the safety of food packaging materials or contact materials should be given more attention.

## 3. Materials and Methods

### 3.1. Reagents and Chemicals

Tert-butylhydroquinone (TBHQ, 98%), butylated hydroxyanisole (BHA, 98%), methyl 2-benzoylbenzoate (OMBB, 98%), 2,2-dimethoxy-2-phenylacetophenone (BDK, 99%), 4-methylbenzophenone (4-MBP, 98%), benzyl butyl phthalate (BBP, ≥95%), 2-hydroxy-4-methoxybenzophenone (UV-9, 99%), 2,2′-dihydroxy-4-methoxybenzo phenone (UV-24, 98%), octabenzone (UV-531, >98%), 2-(2-hydroxy-5-methylphenyl) benzotriazole (UV-P, 99.34%), bis (2-ethylhexyl) phthalate (DEHP, 95%) and trioctyl trimellitate (TOTM, 98%) of analytical grade were purchased from Aladdin Chemical Co. Ltd. (Shanghai, China). Methanol, acetonitrile and isopropanol of HPLC grade were purchased from Tedia Company (Fairfield, CT, USA). Primary secondary amine (PSA, 40–60 μm), graphitized carbon black (GCB, 120–400 mesh) and octadecylsilyl (C18, 40–60 μm) were purchased from Agela (Tianjin, China). Analytical grade anhydrous magnesium sulfate (MgSO_4_) was obtained from Chronchem (Chengdou, Sichuan, China). The ultrapure water used in all experiments was obtained from a Millipore Simplicity purification system (Millipore, Milford, MA, USA).

Individual standard solutions (2000 μg/mL) of twelve target compounds were dissolved in methanol. The mixed stock standard solutions and working solutions were prepared before use by taking certain amounts of each individual standard solutions and diluting with HPLC grade methanol. All solutions were stored at 4 °C in amber glass vessels.

### 3.2. Instruments and SFC System

Chromatographic analysis and separation were carried out by using Nexera UC SFC system (Shimadzu, Japan) with an SPD-20A UV detector. Furthermore, the system was equipped with a column manager, a convergence manager controlling the pressure of supercritical carbon dioxide, an auto-sampler (5 μL loop for injection), an online degasser and a backpressure regulator (BPR). With an LC-20ADXR pump, the modifier could be delivered and then mixed with supercritical carbon dioxide. Lab Solutions Ver5.8 was used for instrument control, data collection and processing.

The final SFC conditions were as follows: A Thermo Scientific^TM^ Acclaim^TM^ 120 C18 (5 μm, 4.6 mm × 250 mm) column was used to separate twelve target analytes. The mobile phase comprised of supercritical CO_2_ (A) and methanol (B). The gradient elution program was set as follows: 0–5 min, 5% B; 5–10 min, 3% B. The flow rate was maintained at 1.4 mL/min. The column temperature was set at 40 °C and the backpressure was 10 MPa. The injection volume was 5 μL. The UV detection wavelength was 220 nm.

### 3.3. Sample Collection and Preparation

All edible vegetable oil samples (extra virgin olive oil, peanut oil, blend oil and sunflower oil) used in this study were purchased randomly from local supermarkets (Hangzhou, China) and stored at room temperature. The general QuEChERS methodology was shown in Figure 1. 0.4 g of oil sample (accurate to 0.1 mg) was weighed in a glass centrifuge tube (10 mL), and 4 mL of acetonitrile was added to the tube. Method optimization and recovery assays were carried out by adding the appropriate mixed standard solution to blank samples in this step. Then, natural samples or spiked samples were vortexed for 3 min and centrifuged at 8000 rpm for 5 min. Afterwards, 2.0 mL of supernatant was transferred into a new glass centrifuge tube containing 800 mg MgSO_4_ and 40 mg PSA, vortexed for 3 min, followed by centrifugation at 8000 rpm for 5 min. Finally, the resulting supernatant was filtered through a 0.22 μm organic membrane into a glass auto-sampler vial for SFC analysis. The use of plastic products was avoided to prevent extra interference during the experiment.

## 4. Conclusions

In the present study, a rapid, simple, green and reliable method was developed and validated for simultaneous determination of twelve chemical additives in edible vegetable oils using a modified QuEChERS procedure followed by SFC. Various parameters such as sorbents, dosage of MgSO_4_, modifier selection, column temperature, flow rate and backpressure were investigated to obtain the optimum experimental conditions. Under the optimal conditions, twelve chemical additives were separated within 10 min, and the consumption of organic solvent was minimized. Satisfactory method validation results were achieved and demonstrated that the proposed method can be regarded as a useful alternative and a complementary method for the routine analysis of additives in edible vegetable oils. Furthermore, the QuEChERS-SFC method is more time-saving, simplified and environmentally friendly. In general, this method provides an efficient procedure for reducing the costs and work involved in the quality control of edible vegetable oils and may have a potential application in other packaged food samples.

## Figures and Tables

**Table 1:** Information of the 12 chemical additives.

Compound	Abbreviation	Category	CAS Number	Molecular Weight	Chemical Structure
tert-butylhydroquinone	TBHQ	antioxidant	1948-33-0	166.22	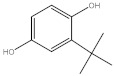
butylated hydroxyanisole	BHA	antioxidant	25013-16-5	180.24	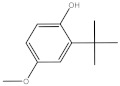
methyl 2-benzoylbenzoate	OMBB	photoinitiator	606-28-0	240.25	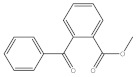
2,2-dimethoxy-2-phenylacetophenone	BDK	photoinitiator	24650-42-8	256.30	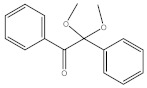
4-methylbenzophenone	4-MBP	photoinitiator	134-84-9	196.24	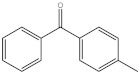
benzyl butyl phthalate	BBP	plasticizer	85-68-7	312.36	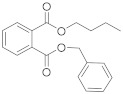
bis (2-ethylhexyl) phthalate	DEHP	plasticizer	117-81-7	390.56	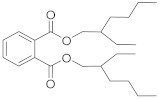
trioctyl trimellitate	TOTM	plasticizer	3319-31-1	546.78	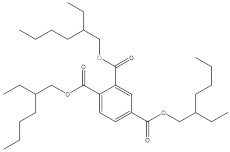
2-hydroxy-4-methoxybenzophenone	UV-9	ultraviolet absorber	131-57-7	228.25	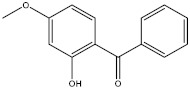
2,2′-dihydroxy-4-methoxybenzophenone	UV-24	ultraviolet absorber	131-53-3	244.24	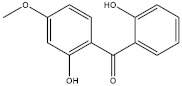
octabenzone	UV-531	ultraviolet absorber	1843-05-6	326.43	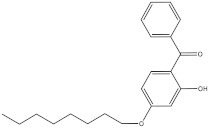
2-(2-hydroxy-5-methylphenyl) benzotriazole	UV-P	ultraviolet absorber	2440-22-4	225.25	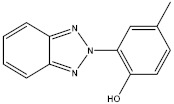

**Table 2:** Linear range, calibration curve, correlation coefficient, LOD, LOQ and matrix effect of twelve polymer additives analyzed by SFC.

Compound	Linear Range (μg/mL)	Calibration Curve	Correlation Coefficient (r)	LOD (μg/mL)	LOQ (μg/mL)	ME (%)
TBHQ	0.50–20.0	y = 2576 x − 95.42	0.99974	0.15	0.50	−3.34
BHA	0.20–20.0	y = 3250 x + 281.71	0.99981	0.06	0.20	+6.70
OMBB	0.20–20.0	y = 6288 x − 216.99	0.99996	0.05	0.15	+2.16
BDK	0.50–20.0	y = 2549 x − 351.41	0.99980	0.09	0.32	−1.85
4-MBP	0.20–20.0	y = 4100 x − 349.61	0.99994	0.06	0.20	−4.30
BBP	0.50–20.0	y = 2666 x + 20.78	0.99908	0.09	0.30	+3.17
UV-9	0.20–20.0	y = 5454 x − 280.37	0.99968	0.05	0.17	+6.19
UV-24	0.20–20.0	y = 6665 x − 54.67	0.99961	0.05	0.18	+5.76
DEHP	0.50–20.0	y = 2271 x − 181.52	0.99985	0.12	0.39	+3.37
UV-P	0.20–20.0	y = 8885 x − 574.34	0.99955	0.05	0.17	+4.57
UV-531	0.50–20.0	y = 3790 x − 65.64	0.99954	0.13	0.43	+1.69
TOTM	0.50–20.0	y = 2193 x + 127.14	0.99907	0.15	0.50	+0.23

**Table 3:** Recoveries and precision in blank edible vegetable oil samples (*n* = 6).

Compound	Spiked Level (μg/g)	Average Recovery (%)	RSD (%)
TBHQ	1	98.5	3.8
	5	98.7	2.1
	10	99.7	3.4
BHA	1	98.9	4.0
	5	98.9	1.4
	10	95.6	0.3
OMBB	1	96.8	6.7
	5	96.7	2.1
	10	96.5	1.0
BDK	1	94.9	7.8
	5	95.6	2.5
	10	84.7	3.9
4-MBP	1	90.0	5.9
	5	90.9	2.3
	10	97.2	0.9
BBP	1	88.0	7.0
	5	96.6	4.4
	10	103.6	0.6
UV-9	1	85.8	1.2
	5	93.5	1.9
	10	101.5	1.7
UV-24	1	83.5	3.4
	5	97.1	4.9
	10	98.9	5.2
DEHP	1	80.3	7.2
	5	84.3	4.0
	10	106.4	1.7
UV-P	1	81.0	1.7
	5	81.1	1.2
	10	83.1	2.2
UV-531	1	78.7	8.5
	5	79.9	7.5
	10	72.7	1.3
TOTM	1	61.6	9.9
	5	61.0	0.6
	10	60.9	7.2

**Table 4:** Analysis of natural samples by QuEChERS-SFC method (*n* = 3).

Samples	Additives Content (μg/g)											
	TBHQ	BHA	OMBB	BDK	4-MBP	BBP	UV-9	UV-24	DEHP	UV-P	UV-531	TOTM
extra virgin olive oil 1	ND ^a^	ND	ND	ND	ND	ND	ND	ND	ND	ND	ND	ND
extra virgin olive oil 2	ND	8.21	ND	ND	ND	ND	ND	ND	ND	ND	ND	ND
peanut oil 1	ND	ND	ND	ND	ND	ND	ND	ND	ND	ND	ND	ND
peanut oil 2	ND	ND	ND	ND	ND	ND	ND	ND	ND	ND	ND	ND
blend oil 1	ND	ND	4.04	ND	ND	ND	ND	ND	ND	ND	ND	ND
blend oil 2	ND	ND	ND	4.47	ND	ND	ND	ND	ND	ND	ND	ND
sunflower oil	ND	ND	ND	ND	ND	ND	ND	ND	ND	ND	ND	ND

ND ^a^: not detected (<LOD).

## Sample Availability

Samples of the compounds are available from the authors.
